# Predictors of physical activity behavior change based on the current stage of change—an analysis of young people from Hawai’i

**DOI:** 10.1007/s10865-021-00255-5

**Published:** 2021-08-27

**Authors:** Eliane S. Engels, Claudio R. Nigg, Anne K. Reimers

**Affiliations:** 1 Friedrich-Alexander-University Erlangen-Nuremberg, Gebbertstr. 123b, 91052 Erlangen, Germany; 2 University of Bern, Bremgartenstrasse 145, 3012 Bern, Switzerland

**Keywords:** Psycho-social predictors, Behavior change, Physical activity, Stage of change, Transtheoretical model

## Abstract

This study investigated the corresponding change between psycho-social predictors and physical activity (PA) behavior and if these relationships were dependent on the stages of change from the Transtheoretical Model in Minority American adolescents. We conducted a longitudinal field study with N = 357 students aged 13–18 years (M = 14.24 years, SD = 0.88); predominantly Filipino (61.2%) using a test–retest design assessing psycho-social PA predictors (enjoyment, self-efficacy, family support, friends’ support, knowledge, stage of change) and moderate-to-vigorous physical activity (MVPA) at two time points over six months. Hierarchical regression results indicated that a positive change of enjoyment, knowledge about PA and family support predicted a change of MVPA, independently of stage. The time-varying covariation showed the importance of the current stage of change for enjoyment, self-efficacy and support of friends for a change of MVPA. Overall, our findings suggest that an individual’s current stage of change should be considered to determine individually appropriate starting points and goals for designing interventions to promote PA among Minority American adolescents.

## Introduction

Only 22.5% of girls and 27% of boys aged 12–15 years in the US meet the WHO (World Health Organization) recommendation of 60 min of moderate-to-vigorous physical activity (MVPA) per day (Bull et al., 2020; Fakhouri et al., 2014). Regular MVPA is associated with numerous positive effects on physical and mental health such as prevention of cardiovascular diseases or positive influence on well-being (Janssen, 2007; Warburton & Bredin, 2017). Filipino-American adolescents show higher levels of health impairment and inactivity compared to other ethnicities of the same age (Javier, Huffman, & Mendoza, 2007; C. Nigg et al., 2011). Further, Filipinos are the second largest Asian-American and Pacific-Islander subpopulation in the USA and underrepresented in research regarding physical activity (PA) promotion (Fleary et al., 2018).

The period of adolescence is generally an important time to establish a healthy lifestyle, which is why physical inactivity is even more of a public health problem (Mulye et al., 2009; Nelson & Gordon-Larsen, 2006). Thus, the question arises on how MVPA can be effectively promoted in young people. Evidence- and theory-based (Downs et al., 2013), specific predictors of change in PA behavior (Rhodes & Quinlan, 2015) and intention-based models have been recommended to understand and promote PA behavior (Ajzen, 1991). A systematic review showed that intention is an important predictor of PA change at an intrapersonal level (Rhodes & Quinlan, 2015). Intention describes the general motivation and readiness of a person to be or become physically active (Ajzen, 1991). Rhodes and Quinlan (2015) emphasize the importance of the intention concept for the individual-level motivation for natural PA change. Furthermore, besides behavior itself, intention differentiates those in different stages of PA adoption (C. R. Nigg, 2005) and is important in distinguishing between those who maintain PA and those who stop PA (Amireault et al., 2013).

The Transtheoretical Model (TTM) (Prochaska & Velicer, 1997) is one of the most common intention-based stage models to describe changes of PA behavior (Marshall & Biddle, 2001; Prochaska & Velicer, 1997). The process of behavior change is divided into five discrete stages. People at the first stage (*precontemplation*) do not meet the PA guidelines and have no intention of doing so. Reasons might be that these people have no or too little information about the negative consequences of physical inactivity. At the second stage (*contemplation*), people also do not meet the PA guidelines, but intend to do so within the next six months. They consider the pros and cons of being active and are considering changing their behavior. If a person intends to engage in regular PA within the next month, then the stage of *preparation* is reached. The person is now ready for regular PA and is actively planning to change behavior. If a person starts to be physically active on a regular basis but has done so for less than six months, he or she is in the *action* stage. If a person continues to successfully maintain this PA behavior for six months or more, the fifth stage (*maintenance*) is reached (Prochaska & Velicer, 1997).

There are numerous studies on the validity and applicability of the PA stages of change (Farmanbar et al., 2009; Lee et al., 2001; Marshall & Biddle, 2001). Most studies examined whether the stages can be differentiated according to PA intensity such as discrimination between stages (C. R. Nigg, 2005). Schumann et al. (2002) have shown that different intensity levels of PA can be distinguished according to the stages of change. Lee et al. (2001) showed validity for the stages of motivational readiness regarding PA behavior among North American adolescents. Adolescents at the earlier stages reported lower PA levels than those at advanced stages. The study of Lippke et al. (2009) also supported the stage model, and indicated that psychosocial variables discriminate between stages at least as well as temporal stage definitions. Fleary et al. (2018) provided evidence about the validity of the TTM stages of change for Filipino and other Asian-American and Pacific Islander adolescents. Marshall and Biddle (2001) conducted a meta-analysis on the applicability of the TTM. In general, their results support the application of the TTM, since the core constructs (self-efficacy, pros and cons and processes of change) differ from stage to stage. Further, the TTM variables have been shown to be invariant by sex, age, ethnicities and over time (Geller et al., 2012; Paxton et al., 2008). Longitudinal application of the TTM showed that the model can serve as a framework to understand adolescent exercise behavior (C. R. Nigg, 2001; C. R. Nigg et al., 2019).

The predictors of change in PA behavior in general have been mainly addressed by intervention studies focusing on PA promotion (Dishman et al., 2005; Lewis et al., 2002; C. R. Nigg et al., 2019; Woods et al., 2002). A review of intervention studies showed that the most important factors of change in PA stages were behavioral processes of change (modification of behavior) and self-efficacy (belief in own abilities) (Lewis et al., 2002). Findings for enjoyment (fun being active) and social support (support from family or friends) were varying due to different measures and statistical procedures (Lewis et al., 2002). Most of these studies included folks who were at the later stages of the TTM. To examine predictors of change over time requires analyses of naturally-occurring changes, because of a greater reach of diverse people regarding stages, ethnic, economic and health than experimental studies that often result in selection biases of highly motivated people and low diverse people (Glasgow et al., 1999).

Longitudinal studies predominantly indicate that social cognitive factors such as self-efficacy and social support are central factors for the prediction of PA behavior change in general (Dishman et al., 2017; Jekauc et al., 2019; Kahn et al., 2002; Lewis et al., 2002; C. R. Nigg, 2001; Rhodes & Quinlan, 2015). Findings regarding enjoyment and knowledge about PA (knowing the benefits of PA) are inconsistent in terms of PA behavior change (Dishman et al., 2017; Jekauc et al., 2019; Kahn et al., 2002; Lewis et al., 2002; C. R. Nigg, 2001; Rhodes & Quinlan, 2015). These inconsistent findings indicate that predictors could have different meanings for predicting adoption and maintenance (D. M. Williams et al., 2008).

A positive affective response to moderate PA has been identified as a correlate of increased PA: Rhodes and Quinlan (2015) conducted a systematic review regarding predictors of PA change among adults using observational designs. Overall, affective judgments (i.e., enjoyment and pleasure from PA) was a reliable correlate of PA adoption. Another study showed that enjoyment is an important factor for the maintenance of PA behavior (Woods et al., 2012). Enjoyment can predict if someone exercises regularly, as the likelihood of exercising increases in relation to the anticipation of positive emotions and therefore can be seen as an antecedent of PA (Mullen et al., 2011; D. M. Williams et al., 2008). Enjoyment can also be seen as a consequence of regular PA (Rovniak et al., 2002). According to the *Broaden-and-Build Theory of Positive Emotions* (Fredrickson, 2004), enjoyment behaves like a positive upward spiral: the more PA, the more enjoyment and so on. Moreover, enjoyment was identified as a large correlate of overall PA change (Ingledew et al., 1998). However, there are also contrary findings regarding behavior change. Over a longer period from childhood to adolescents, change in enjoyment was unrelated to change in PA (Dishman et al., 2017).

In addition, self-efficacy is also an important and frequently investigated construct in the context of the TTM. A meta-analysis showed a consistent positive relation for self-efficacy with stage of intention (Marshall & Biddle, 2001). Williams et al. (2008) compared psychosocial variables regarding adaption and maintenance of PA behavior of adults and found that self-efficacy predicted maintenance but not adaption of PA behavior. Self-efficacy could explain differences between people who dropped out of PA and those who maintained PA (Amireault et al., 2013). Moreover, PA behavior decreased less from childhood to early adolescence in students who reported less decline in self-efficacy (Dishman et al., 2017).

Overall, social support is an important factor for regular PA of young people (Beets et al., 2006; Cheng et al., 2014; Jekauc et al., 2019; Reimers et al., 2019). Social influence from friends and family, was in several studies identified as correlate of change in PA behavior (Rhodes & Quinlan, 2015). Over a longer period from childhood to adolescents, PA behavior decreased less in students who reported less decline in social support from parents and friends regarding activity (Dishman et al., 2017). Within a study about adolescents in Hawai’i, the majority reported that their parents were the dominant influence on their inactive behavior. However, friend related influences were reported to be more important for PA levels (Geller et al., 2014).

Knowledge about health benefits of PA is necessary to motivate, enable or to form an intention in people to become physically active on a regular basis or to change their behavior (Kahn et al., 2002). However, within the review of Rhodes and Quinlan (2015) there was no study reported that found a meaningful relationship between knowledge and change in PA. Underserved population groups often do not have much knowledge about the positive effects and health benefits of PA (Tessaro, Smith, & Rye, 2005). Therefore, a change of PA knowledge should be examined as a predictor of change especially in the early intention stages to reduce health disparities.

The presented studies show a couple of method-based shortcomings. Most research on PA determinants used cross-sectional or passive prospective designs (Bauman et al., 2012). Results based on between-subject-analysis (inter-individual) cannot deliver adequate information about promotion of intra-individual PA behavior change (Weinstein, 2007). There is a lack of studies that examined the time-varying covariation between predictors and changes in PA (Rhodes & Quinlan, 2015). Manly, studies looked at the predictor at baseline followed by an analysis of the change in PA. However, the concurrent analysis of change in the predictor and its corresponding change in PA is important to understand changes in PA as a consequence of the predictor (Baron & Kenny, 1986). Moreover, to examine predictors of change over time requires longitudinal designs with analyses of naturally-occurring changes, because of a greater reach of diverse people regarding PA stages, ethnical background and health (Glasgow et al., 1999). Additionally, the inconsistent findings in previous studies might be due to different specifications and definitions of change in PA behavior within the studies. For example, in some studies the behavior change refers to the increase or decrease compared to the baseline assessment and in other studies to the adoption and maintenance of PA behavior (Rhodes & Quinlan, 2015). In addition, PA change was examined with different methods of data analysis (e.g., residual scores, time-varying covariation scores, dichotomous change scores around a guideline or difference scores) (Rhodes & Quinlan, 2015). These aspects make it difficult to compare the findings across studies.

Furthermore, the following content-based shortcomings are evident: the stage of change of individuals was not considered in most studies. Enjoyment, self-efficacy, social support and knowledge were mentioned as important factors predicting PA behavior change without taking the stage of the participants into account (Dishman et al., 2017; Kahn et al., 2002; Lewis et al., 2002; Rhodes & Quinlan, 2015). Certainly, it seems obvious that it makes a difference if someone is not interested in becoming physically active at all, someone is already planning to become physically active or someone is already active from time to time but does not maintain regular PA. Probably, this is an important issue for targeting and individually tailoring PA promotion to the readiness of the individual.

In summary, theory driven investigation of predictors for behavioral change considering the current PA stage, are still lacking. From a methodological perspective studies are necessary which conduct time-varying covariation among predictors (change–change) (Rhodes & Quinlan, 2015).

The purpose of the present study was to investigate a change in predictor variables and a corresponding change in PA behavior (time-varying covariation between predictors and changes in PA). Based on the mentioned methodological limitations and content-based gaps in previous studies, the aims of the current study were to investigate (1) psycho-social predictors of change in PA behavior (enjoyment, self-efficacy, family support, friends’ support, knowledge) in adolescents and (2) the differences between psycho-social predictors of change depending on the current stage of change. Findings could help determine starting points for interventions and to develop individually tailored interventions for the specific stage of change (Fleary et al., 2018).

## Methods

### Sample

The total sample consisted of *N* = 357 students from one Oahu public high school in the state of Hawai ‘i. Due to power considerations, two independent subsamples were used. The first sample was recruited in 2016–2017 *n*_*1*_ = 132 students and the second sample was recruited in 2017–2018 *n*_*2*_ = 225 students. Demographic comparisons revealed that the samples were similar with respect to age, sex/gender and ethnicity (*p* > 0.05). Participants were aged 13 to 18 years (*M* = 14.24 years, *SD* = 0.88) with the majority being female (71.8%) and primarily Filipinos, however other ethnic groups were represented as well: Filipino (61.2%), Asian (13.5%), Native Hawai’ian or other Pacific Islander (18.5%), Caucasian (2.6%), Japanese (1.8%), Hispanic/Latino (1.5%), Korean (0.3%), Chinese (0.3%).

### Design and procedure

We conducted a longitudinal field study over six months using a test–retest design and measured all variables using a survey at two time points (baseline and follow-up). Whereby 62 respondents had not completed the second measurement. The University of Hawai'i IRB and the Hawai'i Department of Education approved all methods and procedures for the study. Parental consent and participant consent were obtained for all students prior to participation.

### Measures

We measured the following PA related constructs: enjoyment, self-efficacy, social support of family, social support of friends and knowledge about PA (Thompson & Nigg, 2020). Enjoyment is related to the affective experience of PA and was measured by one single item (“I enjoy doing physical activity”) using a 5-point rating scale from 1 (*disagree a lot*), 2 (*disagree a little*), 3 (*neutral*), 4 (*agree a little*) and 5 (*agree a lot*). Self-efficacy describes the confidence to do PA given specific barriers and was assessed via six items (e.g. “I am sure I can participate in regular physical activity when I feel I don't have the time”). Responses were rated on a 5-point-rating scale ranging from 1 (*not at all confident*), 2 (*not very confident*), 3 (*moderately confident*), 4 (*very confident*) to 5 (*completely confident*). The self-efficacy scale provided a good model fit and factorial invariance across age, sex and ethnicity groups in Hawai'i (Paxton et al., 2008). The scale showed internally consistency (*α* = 0.85) for adults living in Hawai’i (Paxton et al., 2008). Social support refers to the perceived support of friends and family members to engage in PA. Social support was assessed with eight items (e.g. “Do your friends tell you that you are doing well in physical activities or sports?”) using a 5-point rating scale (0 = “never”, 1.5 = 1 to 2 days”, 3.5 = 3 to 4 days”, 5.5 = “5 to 6 days” and 7 = “every day”). Answers were recoded so that numeric values best represent a persons’ social support in days per week. Face validity and internal consistency have been reported (Najimi & Ghaffari, 2013). We used the scale means for all constructs that were measured by several items. The item to assess PA knowledge was based on the content of the Waipahu HART curriculum (Geller et al., 2014). Three dichotomous true/false questions were used to address intensities, PA types and recommendations (e.g. “Clearing the table uses the same amount of energy as playing soccer”). Questions answered correctly were summed to obtain a score. Experts who were familiar with the HART curriculum confirmed face validity.

PA stages of change were operationalized as an individual’s readiness to participate in regular PA. The stages are based on the Transtheoretical Model (Prochaska & Velicer, 1997; Schumann et al., 2002). Individuals were asked about participating in sixty minutes per day of MVPA at least five days or more a week, with the activity making the heart beat faster or breathing harder. Answers were classified as precontemplation (not intending to in the next six months), contemplation (intending to in the next six months), preparation (intending to in the next 30 days), action (started regular PA within the last six months) or maintenance (regularly active for six months plus). These PA stages have been validated with adolescents (Calfas et al., 1997) and this population (Fleary et al., 2018).

The outcome variable PA behavior change was operationalized as increases or decreases of MVPA per week from the first to the second time of measurement. PA was assessed using an adapted *Leisure-Time PA Questionnaire* (Godin & Shephard, 1985). Students reported the number of days in a week they performed strenuous PA (makes the heart beat quickly and sweating), moderate PA (makes one tired and sweat a little) and mild PA (takes little effort and does not make one sweat). In addition to frequency, questions regarding duration were asked–on average how many minutes a day the participants spent doing strenuous, moderate and mild activity. Studies have shown evidence that PA behavior can be measured reliable and valid in adolescents (Sallis et al., 1993). As covariates, we included sex/gender,^1^ age and ethnicity of participants and the year of data collection.

### Data analyses

All analyses were performed using SPSS Version 26. Descriptive statistics for all change variables were calculated for the total sample and separately for males and females. Change was calculated using the difference values from time two and time one (Time 2–Time 1), so a positive result indicates an increase. Firstly, for all latent constructs, scale means and standard deviations were calculated for both times of measurement. As quality check for the scales, we computed internal consistencies (Cronbach’s Alpha) for all variables that were measured by several items for both times of measurement. We calculated intra-individual (test–retest) correlations between the first and the second time point (Nonparametric, Spearmans Rho). As preliminary analyses to control for potential third variables, we examined group differences regarding sex/gender, age, ethnicity and year of data collection, by running one-factor analysis of variance (ANOVA) for all change predictors. Furthermore, we conducted ANOVA for a mean value comparison of the four predictors for each stage (planned contrasts) for the first time point. As post-hoc corrections of multiple comparisons, we used Bonferroni correction to avoid the accumulation of the alpha error. Regarding missing values, listwise and pairwise case exclusion was used. To identify relevant relationships between all change variables for males and females, we calculated partial inter-correlations by controlling covariates (age, ethnicity and year of data collection).

To analyze psycho-social predictors of change in PA behavior (research question 1), we conducted a hierarchical regression analysis (HRA) using difference scores of independent and dependent variables (change–change). Using this method, the incremental variance and thus the relevance of each predictor can be measured. This allows to investigate whether the incrementally added predictors explain additional variance in the dependent variable. To examine the differences between psycho-social predictors of change depending on the current stage of change (research question 2), we used a multilevel linear model (MLM) for repeated measurement data with random intercepts and random slopes. Hereby, we could analyze the time varying covariation between predictors and changes in PA as recommend by Rhodes and Quinlan (2015). As covariance type, we have chosen an unstructured covariance matrix, so that the random effect was freely estimated. In addition, we have chosen the Kenward-Roger approximation to improve the precision of the significance tests for the fixed effects. The concurrent analysis of change in the predictor and its corresponding change in PA is important to understand changes in PA as a consequence of the predictor (Baron & Kenny, 1986). To investigate the assumed moderation through intention stage of the relationships between predictors and outcome behavior, we specified interactions with intention stage, predictor and time. We conducted five separate analyses to analyze each predictor (enjoyment, self-efficacy, family support, friends’ support, knowledge), because of the small sample size and for better interpretability.

## Results

### Descriptive statistics and preliminary analyses

Descriptive data is presented in Table 1. Mean values for change between the first and the second measurement point were relatively small for all constructs. Similarly small changes are evident analyzing separately for females and males. The estimates of the intra-individual correlations (Spearmans Rho) showed significant moderate to strong correlations over the period from the first to the second time point (six month), whereby the lowest (moderate) intra-correlation was found for knowledge about PA (*r*_*tt*_ = 0.39) and the strongest correlation for family support (*r*_*tt*_ = 0.65). Internal consistencies (Cronbach’s alpha) ranged from acceptable to good for all scales (self-efficacy *α*_*1*_ = 0.77, *α*_*2*_ = 0.78; family support α_*1*_ = 0.78, *α*_*2*_ = 0.81; friends’ support α_*1*_ = 0.67, *α*_*2*_ = 0.76).

**Table 1:** Mean change values (T2-T1), standard deviations and test–retest correlations for all change variables

	Total sample			Males		Females		Gender diff
	M_change_	SD_change_	r_tt_	M_change_	SD_change_	M_change_	SD_change_	*p*
MVPA (min/week)	44.07	163.22	.52**	67.18	195.64	35.15	148.44	.141
Stage of Change	0.23	1.23	.48**	0.16	1.39	0.26	1.67	.524
Enjoyment	− 0.02	1.05	.50**	− .015	1.13	0.03	1.01	.177
Self-efficacy	0.04	0.78	.60**	− 0.09	0.91	0.09	0.72	.087
Family support	0.08	1.52	.65**	0.11	1.89	0.07	1.35	.853
Friends’ support	0.16	1.42	.53**	0.06	1.64	0.21	1.34	.437
Knowledge	− 0.13	0.87	.39**	− 0.18	0.83	− 0.12	0.87	.569

Distribution of gender and age (absolute frequencies) regarding the initial stages of change groups (T1)									
	Total	Gender		Age in years					
Stage	N	males	females	13	14	15	16	17	18
Precontemplation	32	13	19	5	26	0	0	1	0
Contemplation	80	11	69	10	48	13	7	2	0
Preparation	76	18	58	9	52	6	8	1	0
Action	86	30	56	9	63	8	4	2	0
Maintenance	64	24	40	6	40	4	10	3	1

MVPA = moderate to vigorous physical activity; *r*_*tt*_ = test–retest-correlation; ** the correlation is significant at the level *p* < .01 (two-tailed)

As preliminary analysis, we examined group differences for all relevant behavior change variables (MVPA, intention stage, enjoyment, self-efficacy, support of family, support of friends, PA knowledge) regarding sex/gender, age, ethnicity and year of data collection. No significant differences between female and male participants were found for the analyzed behavior change variables. Regarding the six different age groups, there were significant differences for change of MVPA (*F*(4) = 2.72, *p* = 0.030). The 15-year age group revealed the highest change score and the 17-year age group the lowest. Therefore, in further analyses we controlled for age. The analysis of ethnicity groups showed only significant differences for change of PA knowledge (*F*(8) = 2.67, *p* = 0.008) but not for change of MVPA. The ANOVA for year of data collection revealed significant differences between year 2016/2017 and 2017/2018 for the change of MVPA (*F*(1) = 5.23, *p* = 0.023). Therefore, we controlled for the year of data collection in further analysis. The other behavior change variables did not show any significant differences for the year of data collection.

Results of the primary analysis for mean value comparison of the five PA predictors for each stage (planned contrasts) at the first time point generally showed an increase corresponding to the stages of change. The increase of MVPA levels according to SOC level for both time points confirm that the level of MVPA is associated with SOC level. Mean values and mean differences are reported in Table 2.

**Table 2:** Mean value comparison of PA predictors over stages of change for the first time point (N = 338)

Stage	PA predictors	*N*	*M*	*SD*	*Mean*_diff_	*p*
Precontemplation	Enjoyment	32	3.13	1.18		
Contemplation		80	3.34	0.86	− 0.21	1.000
Preparation		76	3.61	0.80	− 0.27	0.565
Action		86	4.06	0.90	− 0.45	0.011
Maintenance		64	4.55	0.75	− 0.49	0.008
Precontemplation	Self-efficacy	32	2.45	0.78		
Contemplation		80	2.54	0.67	− 0–09	1.000
Preparation		76	2.78	0.78	− 0.23	0.570
Action		86	3.18	0.79	− 0.40	0.009
Maintenance		64	3.61	0.82	− 0.43	0.007
Precontemplation	Family support	32	1.71	1.79		
Contemplation		80	1.66	1.31	0.05	1.000
Preparation		76	1.92	1.36	− 0.25	1.000
Action		86	2.75	1.96	− 0.83	0.025
Maintenance		64	3.87	2.14	− 1.12	0.001
Precontemplation	Friends’ support	32	1.47	1.55		
Contemplation		80	1.42	1.29	0.05	1.000
Preparation		76	1.67	1.10	− 0.25	1.000
Action		86	2.32	1.67	− 0.66	0.038
Maintenance		64	2.83	1.55	− 0–50	0.336
Precontemplation	Knowledge	32	5.03	0.82		
Contemplation		80	4.90	0.74	0.13	1.000
Preparation		76	4.79	0.79	0.11	1.000
Action		86	4.73	0.80	0.06	1.000
Maintenance		64	4.84	0.82	− 0.11	1.000
Precontemplation	MVPA per week (T1)	32	61.25	87.98		
Contemplation		80	77.50	80.42	− 16.25	1.000
Preparation		76	136.18	169.39	− 58.68	0.154
Action		86	221.16	177.26	− 84.98	0.004
Maintenance		64	290.15	176.61	− 68.99	0.058
Precontemplation	MVPA per week (T2)	12	105.83	145.00		
Contemplation		58	104.48	88.34	1.35	1.000
Preparation		81	129.01	102.15	− 24.53	1.000
Action		78	251.41	176.96	− 122.40	0.000
Maintenance		66	360.76	186.90	− 109.35	0.000

PA predictors = Predictors of physical activity; MVPA = Moderate-to-vigorous physical activity; Stage = stage of change; *Mean*_*diff*_ = mean difference compared to the previous stage

Significant partial correlations between change predictors (enjoyment, self-efficacy, family support and friends’ support, stage) and change of MVPA ranged from low to moderate. Only the correlation between change of self-efficacy and change of friends’ support with MVPA change was not significant (Table 3).

**Table 3:** Relationships (partial correlations) between all change variables

Variable	MVPA	Stage	Enjoyment	Self-efficacy	Family support	Friends’ support
MVPA						
Stage	.278**					
Enjoyment	.133*	.186**				
Self-efficacy	.026	.085	.294**			
Family support	.152*	.102	.081	.172**		
Friends’ support	.095	.063	.037	.159**	.183**	
Knowledge	.164**	.063	− .037	− .061	− .098	− .013

Included control variables: year of data collection, age and ethnicity; ** the correlation is significant at the level *p* < .01 (two-tailed); * the correlation is significant at the level *p* < .05 (two-tailed)

### Examination of research questions

To analyze psycho-social predictors of change in PA behavior (research question 1), a HRA was performed with MVPA as the dependent variable and five predictors were entered in five successive steps (enjoyment, self-efficacy, family support, friends’ support, knowledge) (Table 4). From model one to model five the explained variance increased from *R*^2^ = 0.024 to *R*^2^ = 0.084. Indicating that 8.4% of the variance of MVPA change is explained by four predictors, which according to Cohen (1992) corresponds with a calculated *f*^2^ = 0.10 to a small effect. Each predictor contributed significantly to the change in variance of MVPA except for the predictors self-efficacy and friends’ support. A positive change of enjoyment, family support and knowledge regarding PA predicted a positive change of MVPA. The strongest change predictor in the total model was knowledge (*β* = 33.42, *t* = 3.04, *p* = 0.003).

**Table 4:** Hierarchical regression analysis for prediction of change in physical activity behavior (N = 357)

Model	Variable	*R*^2^	Δ*R*^2^	*F*	*df*	*p*	*β*	*t*	*p*
*Male*									
1	Enjoyment	.056	.043	4.34	1	.039	41.14	2.11	.039
2	Enjoyment	.056	.031	2.20	2	.118	40.08	1.87	.012
	Self-efficacy						3.26	0.12	.902
3	Enjoyment	.065	.027	1.70	3	.174	39.85	1.85	.068
	Self-efficacy						− 9.45	− .035	.972
	Family support						10.09	0.85	.397
4	Enjoyment	.077	.025	1.49	4	.214	43.67	1.99	.050
	Self-efficacy						− 4.91	− 0.18	.858
	Family support						8.23	0.69	.495
	Friends’ support						13.01	0.94	.353
5	Enjoyment	.139	.079	2.30	5	.054	44.96	2.11	.038
	Self-efficacy						− 4.17	− 0.16	.875
	Family support						14.55	1.21	.229
	Friends’ support						9.99	0.74	.465
	Knowledge						59.90	2.27	.026
*Female*									
1	Enjoyment	.014	.009	2.85	1	.093	17.95	1.69	.093
2	Enjoyment	.015	.005	1.53	2	.219	18.90	1.75	.082
	Self-efficacy						− 7.26	− 0.47	.637
3	Enjoyment	.047	.032	3.18	3	.025	17.46	1.64	.104
	Self-efficacy						− 12.72	− 0.83	.407
	Family support						19.63	2.52	.012
4	Enjoyment	.056	.037	2.88	4	.024	16.33	1.53	.128
	Self-efficacy						− 15.07	− 0.98	.328
	Family support						17.83	2.27	.024
	Friends’ support						11.05	1.39	.167
5	Enjoyment	.078	.054	3.28	5	.007	16.41	1.55	.123
	Self-efficacy						− 13.50	− 0.89	.377
	Family support						18.06	2.32	.021
	Friends’ support						11.70	1.48	.141
	Knowledge						25.34	2.16	.032

Dependent variable: MVPA = moderate to vigorous physical activity in minutes per week

Furthermore, we analyzed the differences between psycho-social predictors of change depending on the current stage of change (research question 2):

Enjoyment: Regarding the analysis of enjoyment, it was shown that the time-varying covariation of enjoyment and MVPA depends on the intention stage as covariate (*β* = 5.19, *SE* = 2.10, *p* = 0.014). At the next step, a more detailed examination of individual stages (stage as a factor) has shown a negatively significant interaction on the contemplation stage (*β* = −13.78, *SE* = 8.11, *p* = 0.090) and on the preparation stage (*β* = −20.00, *SE* = 7.34, *p* = 0.007). Additionally, we found main effects for stage (*β* = −28.55, *SE* = 12.33, *p* = 0.021) and year (*β* = −109.53, *SE* = 12.03, *p* < 0.001).

Self-efficacy: We found that the time-varying covariation between self-efficacy and MVPA depends on the intention stage (*β* = 5.64, *SE* = 2.47, *p* = 0.023). Further analysis, showed a negatively significant interaction effect for the time-varying covariation on the preparation stage (*β* = −21.80, *SE* = 9.10, *p* = 0.017). Moreover, we found main effects for stage (*β* = 37.24, *SE* = 11.48, *p* = 0.001) and year (*β* = −108.78, *SE* = 12.00, *p* < 0.001).

Family support: The time varying covariation between family support and MVPA has shown to marginally depend on stage (*β* = 3.15, *SE* = 1.61, *p* = 0.051). Regarding the consideration of the different intention stages, we did not find any significant interactions with time and family support. However, we found significant main effects for family support (*β* = 24.61, *SE* = 8.78, *p* = 0.005), time (*β* = 36.20, *SE* = 17.81, *p* = 0.043), stage (*β* = 42.99, *SE* = 7.10, *p* < 0.001) and year (*β* = −117.78, *SE* = 12.02, *p* < 0.001).

Friends’ support: Results showed a significant time-varying covariation for friends’ support and MVPA and that the time varying covariation depends on the stage of individuals (*β* = 6.67, *SE* = 1.99, *p* = 0.001). Moreover we found significant main effects for MVPA, predicted by time (*β* = 46.02, *SE* = 17.45, *p* = 0.009), stage (*β* = 37.44, *SE* = 7.00, *p* < 0.001), year (*β* = -106.06, *SE* = 11.81, *p* < 0.001) and friends’ support (*β* = 41.23, *SE* = 10.86, *p* < 0.001). Within the consideration of the different intention stages, we observed negatively significant interactions for the precontemplation stage (*β* = −30.13, *SE* = 11.44, *p* = 0.009), the contemplation stage (*β* = −26.10, *SE* = 8.28, *p* = 0.002) and the action stage (*β* = −22.34, *SE* = 5.81, *p* < 0.001).

Knowledge: The results did not show a time varying covariation between PA knowledge and MVPA and no interaction effect with stage of change (*β* = 2.90, *SE* = 1.84, *p* = 0.116). However, we found main effects for year (*β* = -111.73, *SE* = 12.30, *p* < 0.001) and stage (*β* = 45.83, *SE* = 13.06, *p* < 0.001). Regarding the different stages, we only found an interaction effect for the time varying covariation between PA knowledge and MVPA for the preparation stage (*β* = −14.19, *SE* = 6.54, *p* = 0.030).

## Discussion

The present study aimed to investigate change in PA behavior of young people from Hawai’i with consideration of their current PA stage of change. Overall, findings indicate that a change regarding knowledge about PA, enjoyment and family support significantly predicted a change of PA behavior in general, independently of the stage. Moreover, findings showed the importance of the current stage of change for enjoyment, self-efficacy and support of friends for a change of PA behavior.

The moderate to strong intra-individual correlations of predictor variables indicate that examined experiences and behaviors were generally stable over a six-month period. The lowest intra-correlation was found for knowledge about PA, which changed the most over time. The strongest correlation and therefore the lowest change was observed for family support, which seems to be more stable over time.

In general, the results support the theoretical assumptions of the TTM and indicate an increase from lower to higher PA stages of change. Only slight deviations from precontemplation to contemplation were observed for the factors family and friends’ support. This is probably because PA behavior is low at these stages and therefore support for it also tends to be low. Previous studies did not show a clear distinction regarding PA behavior (Fleary et al., 2018). However, for knowledge about PA, no increase corresponding to the stage of change was found. This was already evident in our results, as there was no significant correlation between the stage and knowledge. One reason for this could be that the level of knowledge itself is actually not directly related to the level of MVPA, but gains significance once an increase in knowledge has occurred. This is indicated by a significant correlation between change of knowledge and change of MVPA (see Table 3 and findings of HLM).

In our study, a positive change of enjoyment, family support and knowledge predicted a corresponding change of MVPA levels. The strongest change predictor was knowledge about PA: an increased change of knowledge by one unit was associated with an increased level of MVPA of 33 min per week. This is especially interesting with respect to the critique about "rational choice models": according to them, a positive attitude or even knowledge of the positive consequences of PA does not necessarily mean that people are regularly active (Brand, 2006). However, by focusing more closely on the changes, we found that an increase of knowledge is associated with changing MVPA. It can be assumed that the extent of intra-individual changes of knowledge plays an important role, because change of knowledge was as strong predictor. A further explanation could relate to the target group itself, since the participants in our study come from a disadvantaged population group and presumably have less knowledge about PA (Tessaro et al., 2005) and thus more potential for upward change (average score at the first time point = 4.83; potential maximum score = 6.00).

The second strongest predictor for a change in MVPA was a change of enjoyment regarding PA. As the change in enjoyment increased by one unit, MVPA increased by 24 min per week. These results are generally in line with those of Mullen et al. (2011) and Williams et al. (2008), which showed that pleasure is an antecedent of future PA. This finding also corresponds to the Broaden-and-Build Theory of Positive Emotions (Fredrickson, 2004): which posits that more enjoyment is associated with higher levels of PA.

If the change of family support increased by one unit, the level of MVPA increased by 16 min per week. Generally, family plays an important role in traditional Filipino culture and an individual’s behavior is highly influenced by family values (Watkins & Gerong, 1997). In our study, 8.4% of the total variance of behavior change could be explained by change of knowledge about PA, enjoyment and family support. This corresponds to a small effect. The small effect could be reasoned by the fact that we analyzed the data only over six month and that we used difference sores to examine the concurrent change in predictors and its corresponding change in MVPA, as recommended by Baron and Kenny (1986). Therefore, we can conclude that a change in PA is related to a change within the predictors knowledge, enjoyment and family support. Interestingly, a change in self-efficacy did not predict a change of MVPA. This finding is contrary to findings of previous studies (Dishman et al., 2017; Lewis et al., 2002; Marshall & Biddle, 2001). Within the review provided by Lewis et al. (2002) self-efficacy was one of the most important factors for change in PA levels. However, we examined a natural occurring change within six months versus the effects of an intervention as done in the studies reviewed by Lewis et al. (2002). Probably a period of six months is too short to investigate a change without a specific intervention or technique (S. L. Williams & French, 2011).

Overall, findings showed that the relevance of enjoyment, self-efficacy and friends’ support for PA behavior change differed depending on the current stage. Regarding enjoyment, we found a negative interaction with contemplation and preparation stages. An increase of enjoyment was accompanied by a decrease of MVPA and this was stronger at higher stages. However, a suppressor effect is suspected behind these findings, which can result from oversaturation of a model, as indicated by the negative sign. Regarding self-efficacy, we found that an increase in self-efficacy lead to a decrease in MVPA at the preparation stage. This result could also be statistically justified by a suppressor effect. These findings do not support the results of other studies which showed that self-efficacy is mainly relevant for maintenance (D. M. Williams et al., 2008). Therefore, we cannot conclude that self-efficacy is a more relevant predictor of a positive change in PA behavior if adolescents are already at higher stages. Friends’ support, showed a negative interaction for the stages precontemplation, contemplation and action, whereby the extent of negative association with PA was smaller as the stages increased. Therefore, support from friends seems to be particularly important for young people in the action stage. This finding is in line with prior research, which showed that friends had a greater influence on PA than parents (Geller et al., 2014). This study did not differentiate social support between and among friends from social support regarding PA engagement, which could be intersting for future research.

This study combined two approaches for the investigation of PA behavior change: the role of individual stages of change and different predictors of change. The strengths of this study comprise on the one hand the methodology we used to investigate behavioral change. Whereby we followed the recommendations of (Rhodes & Quinlan, 2015) and performed time-varying covariation to examine the corresponding change of predictors and the outcome. On the other hand, in this study we also investigated behavioral change by taking the current stage of an individual which we included as a moderator into account. Due to the specific target group, the results are limited in terms of which group of adolescents MVPA levels are predicted by stage of change and which external variables are associated with MVPA improvement. Moreover, we did not assess access to PA opportunities in the study. However, this would be an important aspect in relation to PA and the opportunities to achieve a meaningful MVPA level.

This study also has some methodological limitations: Our results are limited by self-reported PA, which could bias the findings (Prince et al., 2008). Future research should use device-based measures of PA (e.g. accelerometers, pedometers) to enhance reliability and validity. We only used two measuring points, but to analyze and show changes in behavior more measuring points would be beneficial. Further research should use study designs with at least three measurement points to allow the performance of latent growth models to examine the type of change over time. In addition, suppressor effects appeared in the analysis of stages. Therefore, larger samples should be investigated in future studies in order to investigate complex models with a high number of parameters with a lower probability of error.

## Implications and conclusion

The main aim of this study was to investigate predictors for change of PA behavior in adolescents considering their current intentions to determine suitable starting points and goals for interventions. Findings of our study suggest that a change in enjoyment, a change in family support and a change in knowledge are relevant to change the PA behavior of Filipino youth, independently of the current stage of change. Therefore, positively influencing enjoyment, strengthening family support, and providing knowledge about PA would be a recommended component of any intervention to promote PA among this target group. For example a PA-family-program that involves all family members and includes multi components: increasing knowledge about benefits and effects of regular physical activity, managing mutual support and enhancing pleasure through individually appropriate level of demand. Moreover, our findings indicated the importance of the current stage of change for enjoyment, self-efficacy and support of friends. Adolescents at earlier stages (contemplation and preparation) could profit more from promotion of enjoyment and self-efficacy (e.g. by considering individual preferences, focusing individual progress, and building self-confidence through appropriate challenges) and adolescents at the action stage could profit more from friends’ support to change PA (e.g. by providing team programs for friends). In general, the individual stage of change should therefore be included in the development of appropriate behavioral change interventions to adequately promote PA in adolescents.

## Footnotes

1 We use the term or sex/gender in accordance with the cochrane sex/gender methods group (Demetriou et al., 2019).
